# Emerging Roles of Downstream of Kinase 3 in Cell Signaling

**DOI:** 10.3389/fimmu.2020.566192

**Published:** 2020-09-29

**Authors:** Jia Tong Loh, Joey Kay Hui Teo, Hong-Hwa Lim, Kong-Peng Lam

**Affiliations:** ^1^Bioprocessing Technology Institute, Agency for Science, Technology and Research, Singapore, Singapore; ^2^Singapore Immunology Network, Agency for Science, Technology and Research, Singapore, Singapore; ^3^Institute of Molecular and Cell Biology, Agency for Science, Technology and Research, Singapore, Singapore; ^4^Department of Microbiology and Immunology, Yong Loo Lin School of Medicine, National University of Singapore, Singapore, Singapore; ^5^School of Biological Sciences, College of Science, Nanyang Technological University, Singapore, Singapore

**Keywords:** Dok-3, adaptor, cell signaling, B cells, innate cells

## Abstract

Downstream of kinase (Dok) 3 is a member of the Dok family of adaptor proteins known to regulate signaling pathways downstream of various immunoreceptors. As Dok-3 lacks intrinsic catalytic activity, it functions primarily as a molecular scaffold to facilitate the nucleation of protein complexes in a regulated manner and hence, achieve specificity in directing signaling cascades. Since its discovery, considerable progress has been made toward defining the role of Dok-3 in limiting B cell-receptor signaling. Nonetheless, Dok-3 has since been implicated in the signaling of Toll-like and C-type lectin receptors. Emerging data further demonstrate that Dok-3 can act both as an activator and inhibitor, in lymphoid and non-lymphoid cell types, suggesting Dok-3 involvement in a plethora of signal transduction pathways. In this review, we will focus on the structure and expression profile of Dok-3 and highlight its role during signal transduction in B cells, innate cells as well as in bone and lung tissues.

## Introduction

The specific response of cells to environmental stimuli requires crosstalk between multiple signaling pathways, and such specificity in signaling is achieved through a class of proteins known as adaptors, which link specific protein partners together, in a reversible manner, via their protein binding modules to elicit the appropriate cellular response (1). Downstream of kinase (Dok) is a family of adaptor proteins comprising of seven structurally related members, Dok-1 to -7. Downstream of kinase proteins do not possess any intrinsic catalytic activity, and they function as molecular scaffold to facilitate protein-protein interaction through their distinct protein-binding domains. As their names imply, they play a role downstream of protein tyrosine kinases (PTKs) by connecting these kinases with their downstream effectors, thereby adapting them to various signaling cascades crucial to cellular functions in a spatially and temporally regulated way (2).

Of the seven members in the Dok adaptor family, Dok-1, -2 and -3 are found mainly in hematopoietic cells (3, 4) while the rest are found in other tissues (5, 6). In this review, we will focus on one member of the Dok family adaptor – Dok-3, which was co-discovered by Cong and colleagues as a protein that bound the cytoplasmic PTK Abl (7), and Lemay and colleagues as a heavily tyrosine phosphorylated protein that was associated with the Src homology 2 domain-containing inositol 5′-phosphatase (SHIP) and PTK, Csk (8), and discuss how it functions as a molecular integrator of various immune as well as non-immune signaling pathways.

## Structure of Dok-3

**FIGURE 1:**
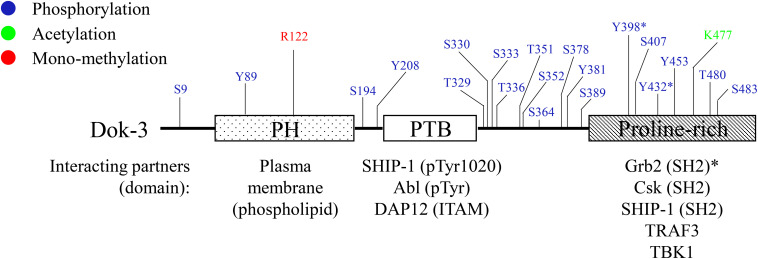
Schematic diagram of human Dok-3. Dok-3 has an amino-terminal pleckstrin homology (PH) and phosphotyrosine binding (PTB) domains, followed by a carboxy-terminal proline-rich region. *Above*, known phosphorylation, acetylation and methylation sites on Dok-3 mapped by mass spectrometry are indicated. *Below*, interaction partners of PH, PTB and proline-rich domains of Dok-3 are shown, together with their respective interacting domains/motifs/amino acid residues. The symbol “*” represents tyrosine residues on Dok-3 which bind specifically to SH2 domain of Grb2.

Downstream of kinase 3 is a relatively small protein comprising of approximately 496 amino acids. At the amino terminus, Dok-3 contains a pleckstrin homology (PH) domain involved in plasma membrane localization via its interaction with membrane phospholipids. Most of the PTKs which phosphorylate Dok-3, as well as its downstream binding partners, are localized to cellular membranes; hence the PH domain is crucial to the membrane localization of Dok-3 for the induction of its function. The PH domain is followed by a central phosphotyrosine binding (PTB) domain which facilitates the binding of Dok-3 to phosphotyrosine-containing consensus motif NPXpY found in partnering proteins. This enables Dok-3 interaction with its upstream PTKs, and the initiation of its function as a scaffold protein to nucleate the formation of multimeric protein complexes. At the carboxyl terminus, there is a proline-rich region with multiple tyrosine residues which can serve as docking sites for SH2-containing proteins upon phosphorylation (2, 7, 8) (Figure 1).

**FIGURE 2:**
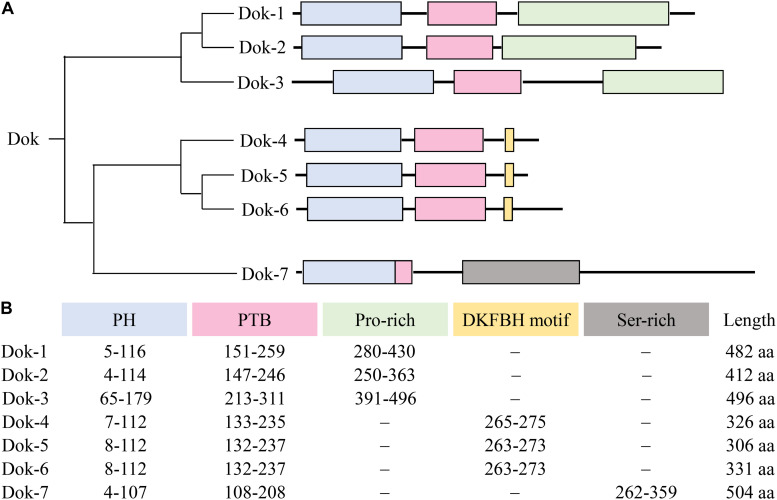
Overall domain architecture of Dok-1-7. **(A)** The Dok family proteins cluster into 3 subgroups, namely Dok-1/2/3, Dok-4/5/6 and Dok-7. All Dok proteins share an amino-terminal PH domain (blue) and a central PTB domain (pink). Dok-1 to -3 consist of a carboxy-terminal proline-rich region (green), Dok-4 to -6 a DKFBH motif (yellow), and Dok-7 a serine-rich region (gray). **(B)** The amino acid residues spanning each domain/motif and the total length of each Dok protein are indicated.

Even though the seven Dok family members share structural similarities characterized by an amino-terminal PH domain followed by a central PTB domain, phylogenetic analysis revealed that they cluster into three different subgroups consisting of Dok-1/2/3, Dok-4/5/6, and Dok-7 respectively (2) (Figure 2). Within the subgroup, the PH and PTB domains of Dok-3 share extensive sequence homology with that of Dok-1 and -2. In particular, a Dok homology (DKH) sequence motif (WPxxxLRxxGxDxxxFxFExGR) has been defined in their PTB domains based on the high sequence similarity observed (9). On the other hand, no conservation in sequence is observed in their carboxy-terminal proline-rich region, possibly accounting for the diverse physiological roles and specificities of Dok-1, -2 and -3 toward different signal-transducing molecules.

## Expression of Dok-3

Unlike Dok-4, -5, and -6 which are preferentially expressed in neural cells and Dok-7 in skeletal muscles (5, 6), Dok-1, -2, and -3 are found mainly in hematopoietic cells and known to be involved in a variety of immunoreceptor signaling pathways (3, 4). Downstream of kinase 3 is abundantly expressed in lymphoid organs such as spleen and bone marrow, but not in the thymus. Correspondingly, high levels of Dok-3 were found in B cells, macrophages, and neutrophils, while undetectable or non-uniform expression of Dok-3 was observed in T cells and their relevant cell lines. Among non-lymphoid tissues, Dok-3 was found predominantly in lungs, moderate levels were detected in the appendix, small intestine, colon, reproductive organs, and urinary bladder, while very low levels were found in other organs like liver, kidney, and brain (7, 8, 10). We will further elaborate on the known physiological role of Dok-3 in each cell type or tissue in the following sections.

## Dok-3 Signaling

Downstream of kinase 3 has been demonstrated to participate in the signaling of B cell-receptor (BCR) in B cells (7, 8, 11–16), and Toll-like receptors (TLR) and C-type lectin receptors (CLR) in innate immune cells such as macrophages (17–20) and neutrophils (21). Since Dok-3 is an adaptor, it has been shown to bind a variety of molecules involved in intracellular signal transduction (Figure 1). Examples of these molecules involved PTKs such as Abl (7), Lyn (14), and Csk (8), serine/threonine kinase TANK-binding kinase (TBK) 1 (20), lipid phosphatase SHIP-1 (8, 12, 13), protein serine/threonine phosphatase 1 (PP1) (21), E3 ubiquitin ligase Cbl (15, 18), and also, other adaptor molecules such as growth factor receptor-bound protein 2 (Grb2) (12–15), TNFR-associated factor (TRAF) 3 (20), TRAF6 (19), DAP12 (17, 22), and Caspase recruitment domain-containing protein (Card) 9 (21).

Downstream of kinase 3 is itself activated by phosphorylation. A number of PTKs have been shown to phosphorylate Dok-3 and these include Abl (7), Src family tyrosine kinases such as Lyn (14) and Src and Tec family of kinases such as Bruton’s tyrosine kinase (BTK) (20). In certain circumstances such as TLR4, 9 and CLR signaling, the phosphorylation of Dok-3 leads to its degradation and de-repression of immune receptor signaling (18–21).

**FIGURE 3:**
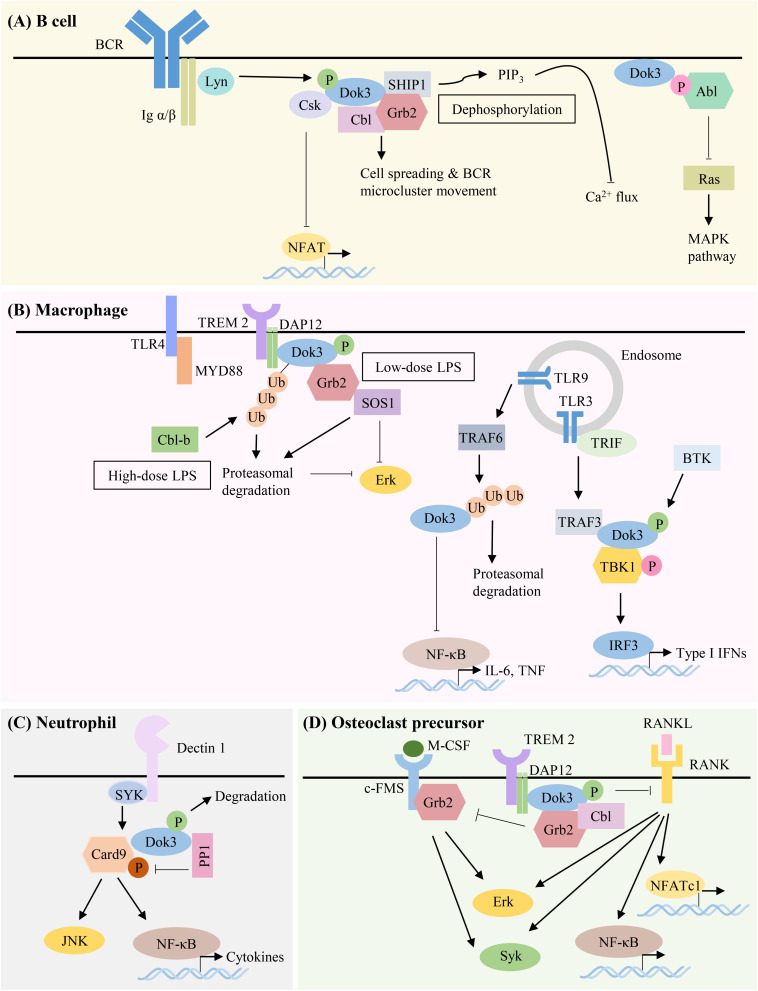
Summary of Dok-3 signaling pathways in B cell, macrophage, neutrophil and osteoclast precursor. Dok-3 functions downstream of multiple receptors including B-cell receptor (BCR), Toll-like receptors (TLRs) and C-type lectin receptors (CLRs) to facilitate the nucleation of distinct signaling molecules and adapt them to regulate cellular functions in a spatially and temporally regulated manner. **(A)** Dok-3 limits BCR signaling by recruiting Grb2/SHIP1 to attenuate calcium flux, interacts with Csk to inhibit NFAT, or sequesters Abl to attenuate MAPK signaling. Dok-3 also associates with Grb2/Cbl to facilitate B cell spreading and transport of BCR microclusters for B cell activation to occur. **(B)** Dok-3 is phosphorylated upon low-dose or ubiquitinated and subsequently degraded upon high-dose LPS stimulation, thereby inhibiting TLR4 signaling in macrophages. Dok-3 also negatively regulates signaling downstream of TLR9. In contrast, Dok-3 is phosphorylated upon TLR3 activation leading to IRF3 induction and IFNβ production. **(C)** Dok-3 also participates in CLR signaling by recruiting PP1 to dephosphorylate Card9 for the attenuation of anti-fungal responses. **(D)** Dok-3 interacts with DAP12 to inhibit osteoclastogenesis.

The multiple kinases that can phosphorylate Dok-3 and the diverse classes of signaling molecules that can interact with Dok-3 reveal the complexity of Dok-3 signaling and highlight its flexibility in orchestrating distinct signaling complexes to achieve specific cellular outcomes (Figure 3 and Table 1).

**TABLE 1:** Roles of Dok-3 and their interacting partners in B cells, macrophages, neutrophils, osteoclasts, osteoblasts, and lung epithelial cells.

Cell type	Activating/inhibitory	Function	Interacting partner	Mode of identification	References
B-cells	Activating	Promotes plasma cell differentiation	–	–	(16)
		Facilitates movement of BCR	Grb2	Immuno-precipitation	(15)
		microclusters for antigen gathering	Cbl		
	Inhibitory	Inhibits BCR signaling	SHIP	Immuno-precipitation, *in vitro* binding assay	(8)
			Csk	Yeast two-hybrid, immuno-precipitation	
			Lck	*In vitro* overexpression	
			Fyn		
			Lyn		
		Inhibits Ras-MAPK pathway to suppress transformation	Abl	Yeast two-hybrid, immuno-precipitation	(7)
		Limits intracellular calcium signaling	Grb2	Affinity Capture-MS	(13)
			Lyn	Affinity Capture-Western	
		Limits production of IgM and proliferative capacity in response to T-cell-independent type I and II antigens	–	–	(11)
Neutrophils	Inhibitory	Inhibits Card9 signaling in response	Card9	Immuno-precipitation	(21)
		to fungal infection	PP1		
Macrophages	Activating	Promotes IFN-β production during	BTK	Immuno-precipitation	(20)
		viral infection	TRAF3		
			TBK1		
	Inhibitory	Inhibits TLR4 signaling	DAP12	Immuno-precipitation	(17, 18)
			Grb2		
			SOS1		
			Cbl-b		
		Negatively regulates IL-6 and TNF-α production during TLR9 signaling	TRAF6	Immuno-precipitation	(19)
		Inhibits malignant transformation together with Dok-1 and Dok-2	–	–	(24)
Osteoblasts and osteoclasts	Activating	Promotes osteoblastogenesis	DAP12	Immuno-precipitation	(22)
	Inhibitory	Inhibits osteoclastogenesis	Grb2		
			Cbl		
Lung epithelial cells	Inhibitory	Inhibits lung tumorigenesis together with Dok-1 and Dok-2	–	–	(26)
		Inhibits pulmonary inflammation together with Dok-1 and Dok-2	–	–	(27)

## Roles of Dok-3

Over the past decades, Dok-3 has generally been implicated in the inhibition of immunoreceptor signaling pathways. However, we now know that this is not always the case. As the roles of Dok-3 in different cell types are starting to be unraveled, accumulating evidence suggests that Dok-3 can function, in a context-dependent manner, as both a positive and negative regulator of signaling pathways, in both immune as well as non-immune cells (Figure 3 and Table 1).

### B Cells

Downstream of kinase 3 was discovered and cloned as a tyrosine-phosphorylated protein which interacts with two inhibitory molecules, SHIP and Csk. SHIP, a 5′ inositol phosphatase implicated in the negative regulation of immunoreceptor signaling, is observed to bind to the PTB domain of Dok-3 via their tyrosine-phosphorylated residues, and this interaction is further facilitated by the association between their SH2 domain and the tyrosine-phosphorylated residues on Dok-3. On the other hand, Csk, a PTK involved in the negative regulation of Src family kinases, is able to contact the tyrosine-phosphorylated residues in the carboxy-terminal region of Dok-3 with their SH2 domain to mediate binding. Since Dok-3 has the tendency to interact with these two inhibitory molecules upon engagement of BCR, and overexpression of Dok-3 inhibited BCR-mediated nuclear factor of activated T-cells (NFAT) activation and Interleukin (IL)-2 secretion in stimulated B cells, this study provided the first line of evidence that Dok-3 functions as a negative regulator of immunoreceptor signaling in B cells (8) (Figure 3A).

The inhibitory function of Dok-3 was further highlighted, when it was cloned by another group by virtue of its interaction with the oncoprotein Abl tyrosine kinase. The PTB domain of Dok-3 binds to the phosphotyrosine residues of Abl in a kinase-dependent manner, and this is important for Abl-mediated tyrosine phosphorylation of Dok-3. Here, overexpression of Dok-3 was found to inhibit Ras-MAP kinase pathway downstream of Abl and suppress its transforming ability *in vivo* (7) (Figure 3A).

Later studies further investigated the physiological relevance of Dok-3 in B cells, and showed its dispensability for early B cell development and the homeostasis of the peripheral B cell populations. Instead, Dok-3 was found to play an inhibitory role in BCR signaling to limit B cell production of IgM antibodies and their proliferative capacity in response to T-cell-independent type I and II antigens, but not to T-cell-dependent antigens. Downstream of kinase 3 knockout B cells exhibited greater induction of NF-κB, JNK and p38 signaling upon BCR stimulation, and their intracellular calcium signaling was also elevated at both low and high dose of stimulations. In the absence of Dok-3, the membrane recruitment of SHIP-1 was unaffected, but its phosphorylation and activation were compromised upon engagement of BCR. Hence, it is likely that Dok-3 exerts its inhibitory effect on BCR signaling through SHIP-1, although the exact mechanism as to how SHIP-1 activation is being controlled by Dok-3 was not further investigated (11).

It was demonstrated in separate studies, that calcium signaling downstream of BCR is attenuated by a Dok-3/Grb2/SHIP-1 module. In resting B cells, Dok-3 is localized to the plasma membrane via its PH domain, while the C-terminal SH3 domain of Grb2 interacts constitutively with the SH3 recognition motif ^1146^PPLPVK^1151^ of SHIP-1 in the cytosol. Upon BCR stimulation, tyrosine phosphorylation of Dok-3 by Src family kinase Lyn promotes the recruitment of cytosolic Grb2/SHIP-1 complex through the phospho-tyrosine (pTyr)-331/SH2 interaction between Dok-3 and Grb2, and PTB/pTyr-1020 within NPXY motif between Dok-3 and SHIP-1. As such, Dok-3 is able to regulate the subcellular localization of SHIP-1 and position it in the vicinity of plasma membrane for dephosphorylation of the lipid phosphoinositol 3,4,5-trisphosphate (PIP3). In this way, it is able to limit plasma membrane recruitment of BCR effector proteins and thus the efficiency of BCR signal transduction including intracellular calcium fluxes (12, 13) (Figure 3A).

The Dok-3/Grb2 module has also been shown to attenuate BCR signaling through the regulation of Lyn-dependent functions. Upon formation of a Dok-3/Grb2 complex at the plasma membrane, the C-terminal SH3 domain of Grb2 facilitates the translocation of the complex to BCR microsignalosomes. Within the microsignalosomes, the proximity of Dok-3/Grb2 complex to Lyn modulates the balance between their inhibitory and activating functions by inhibiting Lyn-dependent Syk phosphorylation and activation, while enhancing Lyn-dependent SHIP activation, thereby negatively regulating BCR signaling (14).

Although majority of the studies focused on the negative regulation of Dok-3 in BCR signaling, Dok-3 has also been reported to play a positive role during B cell activation. Following antigen stimulation of BCR, Dok-3, together with Grb2 and Cbl, form a functional complex at the BCR microcluster to facilitate B cell spreading and the efficient movement of microclusters for antigen gathering. Complex formation is initiated by Grb2 localization to the microcluster via their SH2 domain upon early B cell signaling, and this is required for the subsequent recruitment of Dok-3 and Cbl. Together, these three proteins promote the recruitment of microtubule motor dynein to mediate the transport of BCR microclusters along microtubule network for antigen gathering, as such allowing B cell activation to take place (15) (Figure 3A).

Apart from its role in controlling signaling pathways downstream of BCR in mature B cells, Dok-3 has also been shown to be required for plasma cell differentiation. Although Dok-3 knockout mice had an expansion of germinal center (GC) B and T follicular-helper (T_fh_) cells, the generation of antigen-specific plasma cells and their antibody responses were severely compromised. These Dok-3-deficient B cells were unable to sustain and upregulate the expression of programmed cell death 1 (PD-1) ligand 1 (PDL1) and PD-1 ligand 2 (PDL2) respectively, owing to enhanced calcium signaling due to the lack of negative regulation by Dok-3 as described above. Hence, Dok-3 plays a role in the antigen-driven phase of B-cell differentiation (16).

### Macrophages

Downstream of kinase 3 has been implicated in the negative regulation of TLR4 signaling in bone marrow macrophages, as such preventing excessive inflammation and maintaining endotoxin tolerance upon continuous exposure to lipopolysaccharide (LPS). The PTB domain of Dok-3 binds to the immunoreceptor tyrosine-based activation motif (ITAM) of DAP12, an adaptor protein which couples to various cell surface immunoreceptors such as TLRs. Upon challenge with low-dose LPS, Dok-3 is tyrosine phosphorylated by Src family kinases in a DAP12-dependent manner, resulting in their translocation to the plasma membrane. Subsequently, tyrosine-phosphorylated Dok-3 associates with Grb2 and Son of sevenless homology 1 (SOS1) and sequesters Grb2 from SOS1 to inhibit downstream activation of the Ras-Erk pathway and the production of pro-inflammatory cytokines such as tumor necrosis factor (TNF) α (17). In HEK293 cells, pTyr-398 and pTyr-432 residues in the carboxy-terminal region of Dok-3 mediates its binding to the SH2 domain of Grb2 (23). Upon treatment with higher dosage of LPS, Dok-3 was ubiquitinated by the E3 ligase Cbl-b and degraded, triggering proteosomal degradation of SOS1 and further inhibiting Erk signaling (18). Hence, Dok-3 acts as an indispensable negative regulator of TLR4 signaling through two distinct mechanisms in macrophages. Likewise, Dok-3 is also shown to be a negative regulator of TLR9 signaling as its degradation by TRAF6-mediated ubiquitination is required for TLR 9-mediated IL-6 and TNFα production (19) (Figure 3B).

Contrary to its well-established role as a negative regulator downstream of immunoreceptors, Dok-3 has also been demonstrated to play a positive regulatory role in macrophages during interferon (IFN)-β production in response to influenza virus infection or polyinosinic-polyribocytidylic acid [poly(I:C)] stimulation. Upon stimulation of TLR3, Dok-3 is tyrosine phosphorylated by BTK, and its carboxyl SH2-targeting domain forms a scaffold for the nucleation of TRAF3 and TBK1. As a result, TBK1 is activated by phosphorylation on Ser 172, possibly through autophosphorylation or by an unknown effector kinase, for the downstream induction of IFN regulatory factor (IRF) 3 and hence IFN-β production (20) (Figure 3B).

The compensatory effect of Dok-3 for other closely related Dok family members, Dok-1 and Dok-2, has also been highlighted in the context of macrophages. Studies in mice showed that combined ablation of Dok-1, Dok-2, and Dok-3 promoted the development of histiocytic sarcoma, a malignant proliferation of tissue-resident macrophages. While macrophages lacking Dok-3 alone or Dok-1 and Dok-2 in combination did not show abnormalities, cells with combined loss of Dok-1, Dok-2, and Dok-3 demonstrated enhanced proliferation, suggesting that the Dok proteins mutually compensate for each other’s function and cooperatively limit the proliferation of macrophages in response to macrophage colony-stimulating factor (M-CSF) and granulocyte-macrophage colony-stimulating factor (GM-CSF). However, how ablation of Dok proteins can contribute to the malignant transformation of macrophages, leading to histiocytic sarcoma remains unknown (24). Specifically, it is still not known if Dok-3 plays a direct role downstream of growth factor- or cytokine-receptors.

### Neutrophils

High levels of Dok-3 mRNA have been detected in neutrophils as compared to other myeloid cell types. Here, Dok-3 is shown to negatively regulate the neutrophilic anti-fungal immune responses downstream of CLRs. During steady state, Dok-3 acts as an adaptor to bring Card9 and PP1 in close proximity, enabling the de-phosphorylation of Card9 on threonine residues, thereby suppressing Card9 activity. In this manner, it limits downstream signal transduction to NF-κB and JNK, subsequently inhibiting anti-fungal effector functions such as phagocytosis, pro-inflammatory cytokines production and NETosis in neutrophils. Upon fungal infection, the tyrosine residues on Dok-3 are phosphorylated and Dok-3 is gradually degraded. This degradation leads to an enhancement of fungicidal properties in neutrophils. As a result, mice deficient in the negative regulator Dok-3 had a lower fungal burden in affected organs and were protected from lethal systemic infection with *Candia albicans* (21) (Figure 3C).

### Osteoclasts and Osteoblasts

Downstream of kinase 3 is required for the maintenance of normal bone homeostasis, and Dok-3 knockout mice were found to be osteoporotic, with increased osteoclastogenesis and reduced osteoblastogenesis. In the absence of Dok-3, osteoclast precursors showed increased proliferation in response to M-CSF (22) and increased cell-to-cell fusion of osteoclasts in response to receptor activator of nuclear factor-κB ligand (RANKL) stimulation (25), resulting in an increased differentiation and resorption capacity. Mechanistically, M-CSF and RANKL signaling induced DAP12-dependent phosphorylation of Dok-3, which subsequently forms an inhibitory multimeric complex with Grb2 and Cbl; for the sequestration of Grb2 to limit downstream activation of Syk, NF-κB, Erk, and NFATc1 (Figure 3D). Excessive Erk activation in Dok-3 knockout cells enhances cyclin D1 expression, thereby promoting the proliferation of osteoclast precursors. In addition, Dok-3 was also found to regulate osteoclastogenesis through a DAP12-independent manner, since Dok-3 and DAP12 double knockout mice have normalized bone mass, unlike DAP12 knockout mice which maintained a high bone mass phenotype. On the other hand, Dok-3 promotes osteoblast differentiation and regulates their osteoprotegerin/RANKL ratio to provide another layer of regulation to osteoclast formation. Hence, Dok-3 inhibits osteoclastogenesis and promotes osteoblastogenesis to regulate bone remodeling and prevent osteoporosis (22).

### Lungs

Although the expression of Dok-3 is restricted mainly to hematopoietic cells, abundant amount of Dok-3 was also detected in the non-lymphoid tissue lungs. Notably, mice deficient in Dok-3 were found to develop lung adenocarcinoma, and the effect was more pronounced in mice with compound ablation of Dok-1, -2, and -3, further demonstrating the overlapping and redundancies in function of the family of Dok proteins. Combined loss of Dok-1, -2, and -3 enhanced phosphorylation and activation of Erk and Akt in lung epithelial cells, which promoted the expansion of bronchioalveolar stem cells (BASCs) and their differentiation into alveolar type II (AT2) cells, leading to neoplastic transformation of the lung. Indeed, these *Dok* genes were found to be expressed significantly in the BASC fraction of the lung, further supporting their role in preventing the malignant transformation of these progenitor cells. In particular, loss of *Dok-3* was observed in 7% of primary human lung adenocarcinoma samples from an array-based comparative genomic hybridization dataset, and *Dok-3* was also found to be decreased in lymph node metastases as compared to normal lung tissue, although their reduction was not as significant as that of *Dok-2*, a target of frequent copy-number loss in human lung cancer. Hence, Dok-1, -2, and -3 act in concert as tumor suppressors of the lung, and their compound haploinsufficiency drives lung tumorigenesis in humans (26).

In addition, Dok-1, -2, and -3 function cooperatively to prevent inflammation and maintain homeostasis in the lungs. While Dok-3 single knockout and Dok-1/2 double knockout mice were clinically well, combined ablation of these Dok proteins resulted in pulmonary inflammation, with increased infiltration of macrophages, eosinophils, neutrophils and lymphocytes in the lungs, resulting in an asthma-like airway disorder. An increased production of Th2-type cytokines, including IL-4, -5, and -13, was observed in the bronchoalveolar lavage fluid (BALF) of the triple knockout mice, contributing to inflammation of the airway. Hence, Dok-1, -2, and -3 act cooperatively to negatively regulate pulmonary inflammation (27).

## Conclusion and Future Directions

Adaptor proteins confer specificity in signaling events through their protein binding modules which dictate their binding partners, activity and subcellular localization, thereby regulating protein-protein interactions in a spatially and temporally regulated manner. Downstream of kinase 3 belongs to the Dok family of adaptor proteins, and it has some degree of homology, and as such overlapping functions, with other Dok members, in particular Dok-1 and -2. However, over the past years, emerging evidence suggests that Dok-3 plays a unique role in regulating cell signaling pathways via interaction with a distinct set of signal transducing molecules. Even though it was initially recognized as a negative regulator of immunoreceptor signaling, Dok-3 was later shown to possess both activating and inhibitory functions, in both lymphoid as well as non-lymphoid tissue. Hence, it is becoming clear that Dok-3 is a complex adaptor associated with a plethora of diverse cellular processes implicated in health and diseases such as cancer, bone homeostasis, immune suppression, as well as anti-viral and -fungal immunity. Since Dok-3 was also found to be expressed in organs like colon, reproductive tract and urinary bladder (10), it is interesting to speculate that it may play important roles in regulating other physiological functions in these organs. Moreover, since Dok-3 is known to interact with a variety of other signaling molecules, it remains to be determined if Dok-3 participates in the signal transduction of other immune receptors. Specifically, Dok-3 binds DAP12, which is itself an adaptor associated with numerous immune receptors such as killer immunoglobulin-like receptor (KIR), NKp44, NKG2C/CD94, and NKG2D (28–31). Hence, it is tempting to speculate that Dok-3 might play a role in natural killer (NK) cell functions as well. Thus, more studies need to be done in future for a greater appreciation and understanding of the roles of Dok-3 in signal transduction in various cell types and cell functions.

**FIGURE 4:**
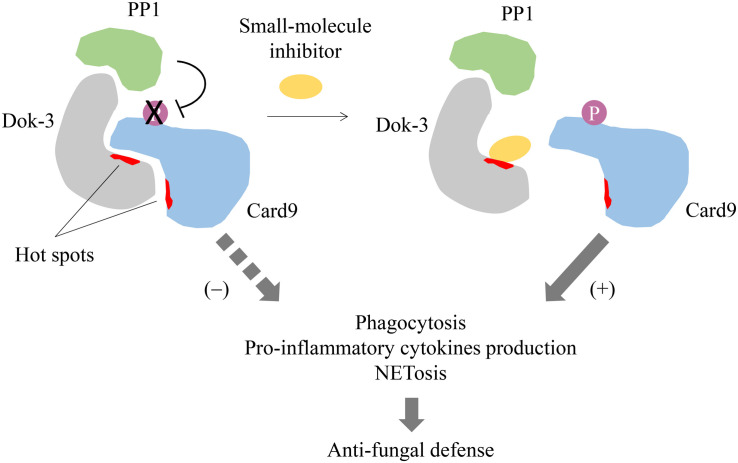
Proposed model for small-molecule inhibition of Dok-3/Card9 binding. Dok-3 recruits Protein phosphatase 1 (PP1) to maintain Card9 in its de-phosphorylated and inactive state in neutrophils. A small-molecule inhibitor targeting hot spots on the Dok-3/Card9 binding interface disrupts interaction between Dok-3 and Card9, allowing Card9 to be phosphorylated and activated, thereby turning on downstream anti-fungal effector functions leading to fungal clearance.

Given the importance of Dok-3 in the mechanistic regulation of cell signaling and its implication in human health and diseases, it will be exciting to see in near future the discovery of drugs which could block the specific interaction of Dok-3 with its various binding partners for prophylaxis or treatment of diseases. For instance, association between Dok-3 and Card9 is pivotal for suppressing Card9 signaling and hence restraining the fungicidal properties of neutrophils such as phagocytosis, pro-inflammatory cytokines release and NETs formation during fungal infection (21). By designing drugs which disrupt the specific interaction between these two molecules, we can potentially remove the brakes on anti-fungal immunity and enhance neutrophil-mediated clearance of fungal pathogens to combat candidaemia (Figure 4). Therefore, Dok-3 represents an important avenue for the development of human therapeutics against various diseases.

Even though adaptor proteins like Dok-3 are essentially viewed as “undruggable,” mainly due to the lack of well-defined binding pockets which modulate protein-protein interactions, recent progress in drug discovery has proven that they are amenable to drug inhibition. High affinity regions termed “hot spots” which drive protein binding have been mapped onto protein-protein interfaces, and these hot spots are particularly adept at binding small-molecule or peptide inhibitors (32, 33). Such inhibitors of adaptors will have the advantage of higher efficacy and increased specificity, and as such reduced toxicity, as compared to conventional kinase inhibitors, which are often non-specific due to the highly conserved nature of the kinase domains (34). Hence, it will be feasible to design and develop inhibitors which disrupt Dok-3 binding with their interacting partners for therapeutic interventions. However, this pursuit is undoubtedly hindered by our limited understanding on the structure of Dok-3. Hot-spots on the binding interfaces between Dok-3 and its protein partners have to be defined to facilitate drug design. As such, it will be necessary for future studies to characterize the protein-protein interaction interfaces on Dok-3, typically through protein-based nuclear magnetic resonance (NMR) or X-ray crystallography, to assess its druggability and for the selection of appropriate drug screening and design strategies. Currently, therapeutic targeting of Dok-3 is still in its infancy, and a combination of biological, chemical and structural approaches is essential to propel its drug discovery forward.

Taken together, Dok-3 is a complex adaptor which forms an integral part of a highly diverse functional network within cells. Although we have gained rich knowledge of Dok-3 signaling and their interacting partners in recent years, its underlying expression and functions in other cell types, tissues and organs remain largely unexplored. With a clear understanding of its signaling mechanisms and physiological roles, there exists an opportunity for the development of Dok-3 inhibitors for clinical intervention of various diseases. Thus, we anticipate in the near future further progress in our understanding of Dok-3 in the field of signal transduction, and the emergence of small-molecule inhibitors of adaptor-binder interactions as a potential therapy to modulate human diseases.

## Author Contributions

JTL and JKHT wrote the manuscript. K-PL conceived the idea. H-HL and K-PL edited and revised the manuscript. All authors contributed to the article and approved the submitted version.

## Conflict of Interest

The authors declare that the research was conducted in the absence of any commercial or financial relationships that could be construed as a potential conflict of interest.
